# Dose-Response Analysis of Chemotactic Signaling Response in *Salmonella typhimurium* LT2 upon Exposure to Cysteine / Cystine Redox Pair

**DOI:** 10.1371/journal.pone.0152815

**Published:** 2016-04-07

**Authors:** Bob T. Rosier, Milena D. Lazova

**Affiliations:** FOM Institute for Atomic and Molecular Physics (AMOLF), Amsterdam, The Netherlands; CSIRO, AUSTRALIA

## Abstract

The chemotaxis system enables motile bacteria to search for an optimum level of environmental factors. *Salmonella typhimurium* senses the amino acid cysteine as an attractant and its oxidized dimeric form, cystine, as a repellent. We investigated the dose-response dependence of changes in chemotactic signaling activity upon exposure to cysteine and cystine of *S*. *typhimurium* LT2 using *in vivo* fluorescence resonance energy transfer (FRET) measurements. The dose-response curve of the attractant response to cysteine had a sigmoidal shape, typical for receptor-ligand interactions. However, in a knockout strain of the chemoreceptor genes *tsr* and *tar*, we detected a repellent response to cysteine solutions, scaling linearly with the logarithm of the cysteine concentration. Interestingly, the magnitude of the repellent response to cystine also showed linear dependence to the logarithm of the cystine concentration. This linear dependence was observed over more than four orders of magnitude, where detection started at nanomolar concentrations. Notably, low concentrations of another oxidized compound, benzoquinone, triggered similar responses. In contrast to *S*. *typhimurium* 14028, where no response to cystine was observed in a knockout strain of chemoreceptor genes *mcpB* and *mcpC*, here we showed that McpB / McpC-independent responses to cystine existed in the strain *S*. *typhimurium* LT2 even at nanomolar concentrations. Additionally, knocking out *mcpB* and *mcpC* did not affect the linear dose-response dependence, whereas enhanced responses were only observed to solutions that where not pH neutral (>100 μM cystine) in the case of McpC overexpression. We discuss that the linear dependence of the response on the logarithm of cystine concentrations could be a result of a McpB / C-independent redox-sensing pathway that exists in *S*. *typhimurium* LT2. We supported this hypothesis with experiments with defined cysteine / cystine mixed solutions, where a transition from repellent to attractant response occurred depending on the estimated redox potential.

## Introduction

Chemotaxis allows bacteria to navigate in gradients of physiologically relevant stimuli, e.g. moving towards higher concentrations of nutrients [1, 2], lower concentrations of toxins [3], or an optimal value of pH [4], oxygen [5], temperature [6] or redox potential [7]. The chemoeffectors are detected in the extracellular space by transmembrane receptors, forming allosteric complexes associated with intracellular histidine kinase molecules [8, 9]. Chemotaxis responses that do not follow the canonical receptor-kinase interaction response have also been described [10], e.g. carbohydrates are sensed via periplasmic proteins that interact with the receptors [11] or using the phosphoenolpyruvate-dependent carbohydrate phosphotransferase systems [12].

The chemotaxis signaling system has been thoroughly studied in the enteric bacterium *Escherichia coli* and its close relative *Salmonella typhimurium* [9, 13]. In short, chemoreceptors, also called methyl-accepting chemotaxis proteins (MCPs) [1], form homodimers, which in turn assemble into allosteric arrays in the membrane [14]. These arrays are responsible for the high sensitivity and cooperative nature of chemotaxis signaling [15]. The receptor-associated kinase CheA transfers a phosphoryl group to the response regulator CheY and phosphorylated CheY (CheY-P), in turn, interacts with the flagellar motors and alters their rotational bias. The phosphatase CheZ accelerates the dephosphorylation of CheY, allowing rapid termination of the signal. Additionally, the activity of the receptor-kinase complex is feedback-regulated by a pair of enzymes, CheR and CheB, which add and remove, respectively, methyl groups to and from specific glutamyl residues of the chemoreceptors (for review see [16]).

Five chemoreceptor species exist in *E*. *coli*, whereas nine chemoreceptor species exist in *S*. *typhimurium* [14, 17]. MCPs have different substrate specificities, with some responding to multiple chemoeffectors [18, 19]. Conversely, some chemoeffectors are sensed by multiple MCPs [17, 20]. Importantly, there are also chemoreceptor species that respond to stimuli via mechanisms other than ligand binding, e.g. Tar and Tsr sense temperature [6] and Aer senses redox potential [21]. The relative abundance of each chemoreceptor is affected by growth conditions and population density of bacteria [6, 22]. Additionally, chemoreceptors sets can differ between different strains of the same species. For example, uropathogenic *E*. *coli* strains generally lack the ribose / galactose sensing receptor Trg and peptide-sensing receptor Tap [23].

The amino acid *L*-cysteine (Cys) and its oxidized dimeric form *L*-cystine (CySS) are chemoeffectors of opposing sign for the *S*. *typhimurium* 14028 strain [17]. The oxidized (CySS) and reduced (Cys) forms are also sensed by different subsets of chemoreceptors. Lazova et al., [17] showed that the repellent response to CySS is mediated by McpB and McpC, and the attractant response to Cys is mediated by Tsr and Tar [17, 20]. The adaptive recovery upon CySS step stimulation has been shown to be incomplete, and the imperfect adaptation has been suggested to promote spreading in motility-plate chemotaxis assays [17].

Here we investigated the dose-dependent response of the strain *S*. *typhimurium* LT2 to the Cys / CySS redox pair using *in vivo* fluorescence resonance energy transfer (FRET) measurements. The dose-dependent response to the reduced form, Cys, was sigmoidal and Tsr / Tar-dependent. Interestingly, in the Tsr / Tar knockout strain we detected a repellent response to Cys, scaling linearly with the logarithm of the Cys concentration. We then tested the oxidized form, CySS, and observed similar linear scaling of the repellent response. Surprisingly, CySS detection started at nanomolar concentrations and the linear scaling continued over more than four orders of magnitude until the highest tested concentration (*i*.*e*., 500 μM). Notably, at nanomolar concentrations, another oxidized compound–benzoquinone–triggered similar responses. In contrast to previous results in *S*. *typhimurium* 14028 [17], we detected McpB / C-independent responses to CySS in the LT2 strain even at nanomolar concentrations. Knocking out *mcpB* and *mcpC* did not affect the linear dose-response dependence, whereas enhanced responses were only observed to solutions that were not pH neutral (>100 μM CySS) in the case of McpC overexpression. We discuss that the linear dependence of the response on the logarithm of CySS concentrations could be a result of a McpB / C-independent redox-sensing pathway that exists in *S*. *typhimurium* LT2. We supported this hypothesis with experiments with defined Cys / CySS mixed solutions by applying the Nernst Equation and showing a transition from attractant to repellent response depending on the estimated redox potential.

## Materials and Methods

### Bacterial strains and plasmids

All strains and plasmids used in this work are listed in Table 1.

**Table 1:** Strains and plasmids used in this study.

**Strain**	**Relevant genotype**			**Source**
LT2	*Salmonella enterica* serovar Typhimurium strain LT2 (wild type *S*. *typhimurium* LT2)			*Salmonella* Genetic Stock Center (SGSC)
TSS500	LT2 Δ*cheY* Δ*cheZ*			[17]
TSS878	LT2 Δ*tar* Δ*cheY* Δ*cheZ*			[17]
TSS868	LT2 Δ*tsr* Δ*cheY* Δ*cheZ*			[17]
TSS866	LT2 Δ*tar* Δ*tsr* Δ*cheY* Δ*cheZ*			[17]
TSS941	LT2 Δ*mcpB* Δ*cheY* Δ*cheZ*			This work
TSS942	LT2 Δ*mcpC* Δ*cheY* Δ*cheZ*			This work
TSS958	LT2 Δ*mcpB* Δ*mcpC* Δ*cheY* Δ*cheZ*			This work
14028	wild type *S*. *typhimurium* ATCC strain 14028			Rasika Harshey [17]
SM542	14028 Δ*mcpB* Δ*mcpC*			Rasika Harshey [17]
TSS643	LT2 Δ*aer* Δ*mcpC*			Kelly Hughes
**Plasmid**	**Gene(s)**	**Resistance**	**Induction**	**Source**
pVS88	*cheZ-ecfp / cheY-eyfp*	ampicillin	IPTG	[24]
pKG116	cloning vector	chloramphenicol	sodium salicylate	J.S. Parkinson
pBR1	*mcpB*	chloramphenicol	sodium salicylate	This work
pML19	*mcpC*	chloramphenicol	sodium salicylate	This work

CheY-YFP and CheZ-CFP fusions for the FRET experiments were expressed from a plasmid pVS88 [24], induced with 150 μM isopropyl β-D-1-thiogalactopyranoside (IPTG). In the strains used in FRET experiments, except the strains from R. Harshey’s lab and K. Hughes lab (Table 1), chromosomal *cheY* and *cheZ* were deleted in order to prevent a competitive interaction between fluorescent protein fusions CheY-YFP and CheZ-CFP, and wild-type (WT) CheY and CheZ expressed from the chromosome, which could lead to a smaller amplitude of the FRET response [17].

Plasmids for McpB and McpC expression (pBR1 and pML19 respectively) were constructed by PCR amplification of genomic *mcpB* and *mcpC* using primers containing NdeI and BamHI restriction sites for ligating into the same sites on the expression vector pKG116. Induction of protein expression was achieved using 7 μM sodium salicylate (Na Sal).

In-frame deletion of genes in the strains designed for this study was achieved by allele-replacement procedure based on Datsenko and Wanner’s method [25]. The initial deletion step involved an insertion of a cassette that provides kanamycin resistance, and also contains the lethal gene *ccdB* under the control of *L*-rhamnose inducible promoter, allowing the cassette to be removed by positive selection on *L*-rhamnose-minimal plates [26]. *Salmonella*’s resident plasmid pSLT contains a *ccdA ccdB* operon that interferes with the deletion strategy. Thus, pSLT was displaced prior to allele replacements using Kit10 from *Salmonella* Genetics Stock Collection (SGSC): a plasmid pLL6, which is from the same compatibility group as pSLT, was transformed in the strains of interest, pSLT was cured, and pLL6 was subsequently removed using temperature selection [27].

All gene deletions as well as plasmid constructs were confirmed by sequencing.

### Growth conditions

In all experiments cells were grown at 33.5°C to mid-exponential phase (OD_600_ ~ 0.5) in tryptone broth (TB) (1% Bacto tryptone, 0.5% NaCl, pH 7.0), supplemented with appropriate antibiotics and inducers (Table 1). Bacteria were harvested by centrifugation, washed and resuspended twice in motility buffer (10 mM potassium phosphate buffer pH 7.0, 0.1 mM EDTA, 1 μM *L*-methionine, 10 mM lactic acid, pH 7.0), and stored at 4°C 1–5 h prior to the experiment.

### Chemoeffector preparation

Chemoeffector solutions were prepared as follows. A stock solution of 500 mM CySS (*L*-cystine, Calbiochem, 99.1%, with a certified synthetic origin) was prepared in 1 M HCl because of the poor solubility of CySS in water. Working dilutions were prepared in motility buffer. It has been reported previously that CySS dissolved directly in motility buffer (without using HCl) also elicits a repellent response [17]. Cys (*L*-cysteine, Sigma Aldrich, from non-animal source) 100 mM stock and dilutions were prepared directly in motility buffer. The stocks of all other chemoeffectors were made directly in motility buffer. The stock solutions and dilutions of all chemoeffectors were freshly prepared on the day of the FRET experiment (1–3 h prior to the experiment).

### Fluorescence resonance energy transfer (FRET) experiments and data analysis

*In vivo* FRET microscopy on live bacterial populations was performed as described previously [28]. Bacteria, expressing the FRET donor-acceptor pair CheZ-CFP / CheY-YFP, were immobilized on a poly-*L*-lysine–coated microscope coverslip, attached to the top surface of a flow cell [29], and kept under constant flow of motility buffer, generated by a syringe pump (Harvard Apparatus, PHD2000). During the experiment chemoeffectors were added and removed by continuous flow, alternating between solutions using a switch valve (Hamilton). There is a nearly constant delay of ~25 s between the time in which the change of solutions is induced by switching the valve (indicated as time 0 in the figures) and the time when the new solution reaches the bacteria at the flow cell.

FRET microscopy on bacterial populations was performed on an upright microscope (Nikon FN1), equipped with an oil immersion objective (Nikon CFI Plan Fluor, 40x/1.3). The bacteria in the flow cell were illuminated by a metal halide arc lamp with closed-loop feedback (EXFO X-Cite *exacte*) through an excitation bandpass filter (Semrock, FF01-438/24-25) and a dichroic mirror (Semrock, FF458-Di01). The epifluorescent emission was split by a second dichroic mirror (Semrock, FF509-FDi01) into donor (cyan, *C*) and acceptor (yellow, *Y*) channels. The signals from the *C* and *Y* channels, passed through emission bandpass filters Semrock FF01-483/32 and FF01-542/27 respectively, were collected by photon-counting photomultipliers (Hamamatsu H7422P-40). Signal intensities were recorded through a data acquisition card (National Instruments) installed on a PC, running custom-written software.

The ratio *R* between the coverslip background-corrected *Y* and *C* fluorescence signal intensities: *R* = *Y*/*C*, provided an indicator of FRET activity, robust to fluctuations in the intensity of the light. The change in FRET activity upon stimulation, Δ*FRET*, can be expressed as a function of the change in the ratio Δ*R*,

$$\Delta FRET=\frac{R_{pre}+\Delta R-R_{0}}{R_{pre}+\Delta R+|\Delta Y/\Delta C|}-\frac{R_{pre}-R_{0}}{R_{pre}+|\Delta Y/\Delta C|},$$

where *R*_pre_ is the pre-stimulus acceptor to donor ratio, Δ*R* = *R* − *R*_*pre*_ is the ratio change, *R*_0_ is the acceptor to donor ratio in absence of FRET, and |Δ*Y*/Δ*C*| is the constant absolute ratio between the changes in the acceptor and donor signals per FRET pair (for the setup in this study, |Δ*Y*/Δ*C*|≈0.6) [28]. Under the measurement conditions *R*_*pre*_ + |Δ*Y*/Δ*C*| ≫ Δ*R*; thus Δ*FRET*∼Δ*R*. (Δ*FRET* is expressed in arbitrary units of Δ*R* throughout the study).

## Results

### Dose-response dependence of the response to Cys in the strain *S*. *typhimurium* LT2

**Fig 1:**
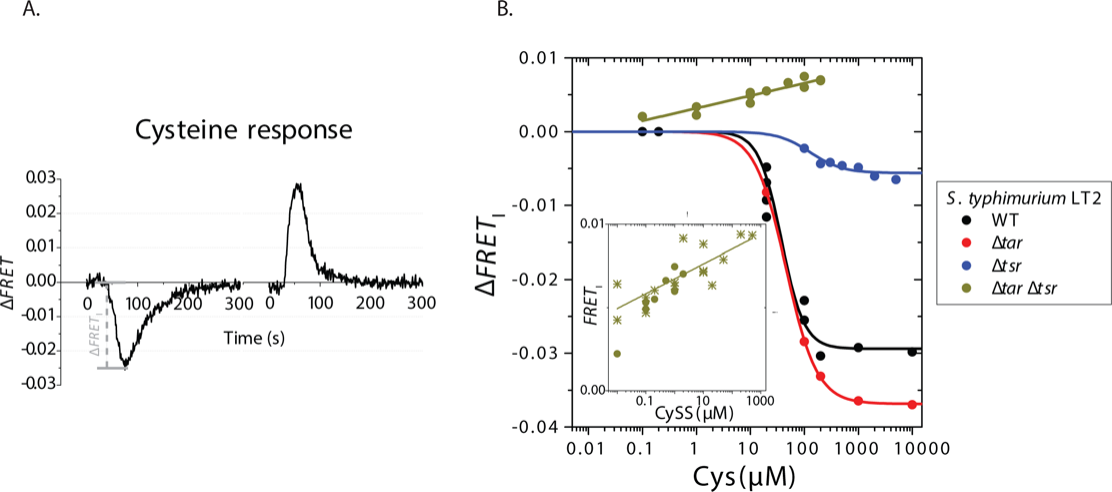
Attractant response to Cys of *S*. *typhimurium* LT2. **(A)** Typical time series of addition (left) and removal (right) of Cys (100 μM) in WT *S*. *typhimurium* LT2. Cys is added and removed at times 0 (see Materials and Methods) **(B)** Initial amplitudes of the FRET response (Δ*FRET*_I_) to Cys of WT, Δ*tar*, Δ*tsr*, and Δ*tar* Δ*tsr* strains are plotted as a function of the Cys concentration. The respective Hill equation fits with half maximum of 38, 46, and 128 μM, and Hill coefficients of 2.1, 1.5, and 1.5 are shown for the first three strains. An apparent linear fit is shown for the Δ*tar* Δ*tsr* strain. **Inset:** Comparison of the measured response of the Δ*tar* Δ*tsr* strain to CySS (stars) and the expected response to CySS upon stimulation with Cys solutions, in which 1% of the Cys is oxidized to CySS.

To characterize the chemotactic signaling response to the reduced form, Cys, we applied sequential steps of Cys with increasing concentrations to populations of *S*. *typhimurium* LT2 cells immobilized in a flow cell. We measured the output of the chemotaxis system using a FRET assay utilizing a donor-acceptor pair between the phosphatase CheZ and the response regulator CheY fused to yellow and cyan fluorescent proteins (YFP and CFP), respectively [24, 28, 30–32]. CheY phosphorylation by CheA and its dephosphorylation by CheZ have equal rates at steady state. Thus, the FRET efficiency is proportional to the concentration of the CheZ⋅CheY-P complex, and this FRET assay provides a measure of CheA activity on time scales longer than the relaxation time of the CheY phosphorylation cycle (~100 ms) [28]. A typical FRET response time series to Cys is shown on Fig 1A (Cys is added and removed at times 0 on the left and right panels respectively; see Materials and Methods). The initial decrease of the FRET level upon addition of Cys indicated an attractant response, which was followed by a perfect (complete) adaptation to the prestimulus level. Upon removal of Cys, a transient increase in the FRET level followed by a perfect adaptation was observed.

Lazova et al [17] showed that the attractant response to Cys in *S*. *typhimurium* 14028 is Tar and Tsr dependent. Here we used another strain of *S*. *typhimurium*, LT2, which is the principal strain for cellular and molecular biology in *Salmonella* [33, 34]. We applied steps of Cys with increasing concentrations and measured the FRET response in order to characterize the dose-response dependence for Cys in wild type, Δ*tar*, Δ*tsr*, and Δ*tar* Δ*tsr S*. *typhimurium* LT2 strains (Fig 1B). The dose-response curve for Cys in wild type *S*. *typhimurium* LT2 showed a sigmoidal shape, with a threshold below 20 μM, half-maximum at ~40 μM and saturation after ~100 μM. The amplitude of the saturating response was greatly diminished in the Δ*tsr* strain, confirming that Tsr is the dominant receptor for Cys in *S*. *typhimurium* LT2. In contrast, knocking out *tar* did not decrease the amplitude of the response. Moreover, the amplitude of the saturating response of the Δ*tar* mutant was slightly higher as compared to WT LT2. The latter observation could be explained by changes in the composition of the receptor clusters formed by multiple receptor species [14]: the deletion of the major receptor Tar would increase the homogeneity of the clusters, which in turn would lead to higher sensitivity and cooperativity of the response, and higher amplitude of the saturating response [24].

However, we confirmed that Tar does have a role in the detection of Cys as an attractant, demonstrated by our experiment with the double knockout Δ*tar* Δ*tsr* strain, which did not show an attractant response to Cys for any of the tested concentrations. Surprisingly, in this strain the FRET levels increased upon stimulation (Fig 1B), indicative for a repellent response to Cys. A plausible explanation for the repellent response to Cys of the Δ*tar* Δ*tsr* cells is that under the aerobic conditions of our experiments, part of the Cys oxidizes to CySS [35]. This effect was only detectable in the Δ*tar* Δ*tsr* strain indicating that as long as Tar or Tsr were present, the attractant response to Cys dominated.

### The magnitude of the response to CySS is proportional to the logarithm of CySS concentration

**Fig 2:**
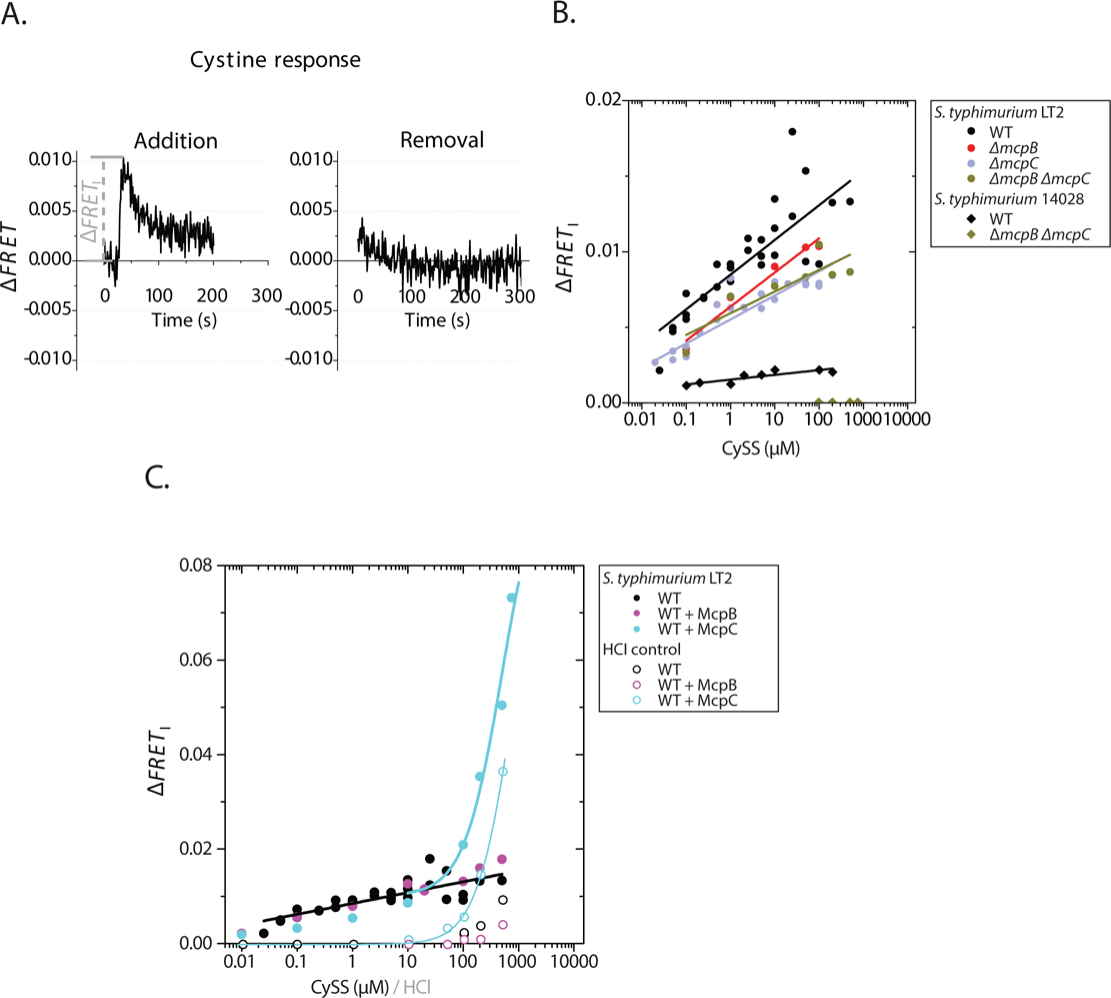
**Repellent response to CySS of *S*. *typhimurium* LT2 and 14028 (A)** Typical time series of addition (*left*) and removal (*right*) of CySS (100 μM) in wild type (WT) *S*. *typhimurium* LT2. CySS is added and removed at times 0. **(B)** Initial amplitudes of the FRET response (Δ*FRET*_I_) of WT *S*. *typhimurium* LT2, Δ*mcpB* LT2, Δ*mcpC* LT2, Δ*mcpB* Δ*mcpC* LT2, WT *S*. *typhimurium* 14028, and Δ*mcpB* Δ*mcpC* 14028 strains are plotted as a function of the CySS concentration. The apparent linear fits for the first 5 strains are plotted. Δ*mcpB* Δ*mcpC* 14028 does not respond to CySS in the tested concentration range. **(C)** Initial amplitudes of the FRET response (Δ*FRET*_I_) of WT *S*. *typhimurium* LT2, LT2 overexpressing McpB, and LT2 overexpressing McpC (see Materials and Methods and Table 1). The open symbols represent controls of stimulating the same strains with HCl with molarity identical to the one used in the CySS solutions. The apparent linear fit for the WT strain (black line) and exponential fits to the response to concentrations of CySS and HCl control ≥ 10 μM (blue curves) are plotted.

CySS, the oxidized molecule in the Cys / CySS redox pair, elicited a repellent response in *S*. *typhimurium* LT2: the FRET ratio transiently decreased, followed by imperfect (partial) adaptation to the prestimulus level (Fig 2A left). Upon removal of CySS, the FRET level returned to the prestimulus level (Fig 2A right). We measured the initial amplitudes of the FRET response to a CySS step increase, Δ*FRET*_*I*_, and plotted them as a function of the added CySS concentration (Fig 2B). We detected responses to CySS for concentrations as low as 20 nM (lower concentrations were also tested, and although responses were present at 1 and 10 nm, they were not plotted because the amplitudes of the responses were close to the detection limit of our FRET assay). For the concentration range 20 nM–500 μM, the amplitude of the response scaled linearly with the logarithm of the CySS concentration.

Changes in pH elicit Tar / Tsr-dependent chemotaxis response in *E*. *coli* [22]. The CySS stock solutions used in our experiments were dissolved in HCl (see Materials and Methods). The tested solutions of CySS were diluted in motility buffer had a pH of 7.0 until reaching a concentration of 100 μM CySS, where the pH started dropping down gradually until reaching a pH of 6.7 at 500 μM CySS. Thus, as a control for pH responses, we tested the response to solutions of HCl that had the same molarity as the HCl used for the respective CySS dilutions (Fig 2C). In the LT2 WT strain, we observed responses to amounts of HCl equivalent to HCl amounts in the >100 μM CySS solutions. However, the amplitudes of the responses were lower than the solution that contained CySS. As expected, no response was detected to the lower HCl concentrations where the pH of the solutions was neutral.

Similar to the response to CySS, the repellent response to the reduced form, Cys, solutions of the Δ*tar* Δ*tsr* strain scaled linearly with the logarithm of Cys concentration (Fig 1). The magnitude of the measured responses to Cys solutions of the Δ*tar* Δ*tsr* strain is very similar to the ones to CySS solutions with 50-times lower concentration (see Fig 1, Inset). This indicates that ~1% of Cys in our Cys solutions might have been oxidized to CySS under the aerobic conditions of our experiments (note that two Cys molecules get oxidized to form one CySS molecule).

### McpB / C knockouts do not affect the shape of CySS dose-response curve in *S*. *typhimurium* LT2 nor does overexpression until 100 μM

The shape of the dose-dependent response to CySS is atypical for receptor-ligand binding interactions, which are characterized by linear mass action kinetics or positively cooperative binding, *i*.*e*. Hill coefficients *n*_H_ ≥ 1 [24, 31, 36, 37]. Fits with a Hill equation of the CySS dose-response data gave Hill coefficients *n*_H_ = 0.50±0.15. A plausible explanation for the atypical shape of the dose-response curve could be that multiple receptors with different affinities were binding CySS [38], or there was a mechanism that did not involve receptor binding.

To test if the atypical shape of the dose-response curve to CySS was due to multiple receptors binding CySS with different affinities, we knocked out and overexpressed McpB and McpC. It was previously shown that these two chemoreceptors of *S*. *typhimurium* sense CySS as a repellent, where deletion of both receptors in *S*. *typhimurium* 14028 completely abolished the repellent response to CySS [17]. If McpB and McpC bind CySS independently with different affinities, the net response might be characterized with apparent negative cooperativity (*i*.*e*. Hill coefficient *n*_H_ < 1), which might explain the shape of the observed dose-response curve. To test this hypothesis, we measured the dependence of Δ*FRET*_I_ on the CySS concentration in Δ*mcpB*, Δ*mcpC*, and Δ*mcpB* Δ*mcpC S*. *typhimurium* LT2. Unexpectedly, the thresholds of CySS response for all tested strains stayed below 100 nM, and a linear dependence of the amplitude of their response with the logarithm of CySS concentration was still observed (Fig 2B). Additionally, overexpressing McpB and McpC did not change the response from the WT, apart from at concentrations at which the negative control with just HCl was triggering a response (*i*.*e*., >100 μM) (Fig 2C). In the case of McpB overexpression, there was no significant difference from the WT. However, when overexpressing McpC, there was an increase in the response to HCl concentrations that decreased the pH with 0.1–0.3, *i*.*e*. control solutions for 100–500 μM CySS, indicating that McpC might have a role in proton / pH detection. As this was not the scope of this research, we leave the question about the role of McpC in pH taxis for future studies.

Our results suggested that a McpB / C-independent mechanism of CySS sensing exists in the strain *S*. *typhimurium* LT2, which might not exist in *S*. *typhimurium* 14028. Due to this unexpected result, we also performed a dose-response analysis of CySS in WT and Δ*mcpB* Δ*mcpC S*. *typhimurium* 14028 strains from our last study [17]. Here we also observed linear scaling of the response with the logarithm of CySS concentration in wild type 14028, starting at concentrations below 100 nM (Fig 1B).

The amplitudes of the CySS responses of 14028 are much smaller than those of LT2. Therefore, despite that we confirmed that the Δ*mcpB* Δ*mcpC S*. *typhimurium* 14028 did not respond to any of the tested concentrations, we considered the possibility that the responses could have been below background level in this and the previous study. In light of this, wild-type *cheY* and *cheZ* are present on the genome of the 14028 strains, in addition to the *cheY-yfp* and *cheZ-cfp* genes on the plasmid for expression of the FRET pair, whereas only CheY-YFP and CheZ-CFP are present in LT2 (see Materials and Methods). Competitive interactions of native and fluorescent protein fusions of CheY and CheZ might explain the smaller amplitudes of the response of the 14028 strains. However, we noticed that the responses of the LT2 Δ*mcpC* and LT2 Δ*mcpB* Δ*mcpC* strains have very similar amplitudes within the whole range of tested concentrations (Fig 2B), whereas Lazova et al. showed a clearly detectable response of the 14028 Δ*mcpC* strain to CySS, but no detectable response of the 14028 Δ*mcpB* Δ*mcpC* strain to CySS [17]. This suggests that the WT *cheY* and *cheZ* on the chromosome on 14028 are not the reason for the absence of the response to CySS in 14028 Δ*mcpB* Δ*mcpC*.

Another explanation of the smaller amplitudes of the response of *S*. *typhimurium* 14028 might be different expression levels of some of the chemotaxis network components between LT2 and 14028 strains.

Although it has been beyond the scope of this study to identify all possible receptors different than McpB and McpC that sense CySS in *S*. *typhimurium* LT2, we suspected that Aer could be such a receptor. Aer is involved in redox sensing [5], and moreover, *aer* is located immediately upstream of *mcpC*. Although the two genes have distinct flagellar class 3 promoters, insertions in *aer* are polar on *mcpC* [17]. We tested the response to CySS of a double knockout Δ*aer* Δ*mcpC* strain (S1 Fig). The Δ*aer* Δ*mcpC* strain showed a repellent response to CySS similar to Δ*mcpC* strain. Note that native *cheY* and *cheZ* are present on the chromosome of the Δ*aer* Δ*mcpC* strain, so direct quantitative comparison of the amplitudes of the response is not trivial.

### The response to another oxidized redox component, benzoquinone, is similar to the CySS response

**Fig 3:**
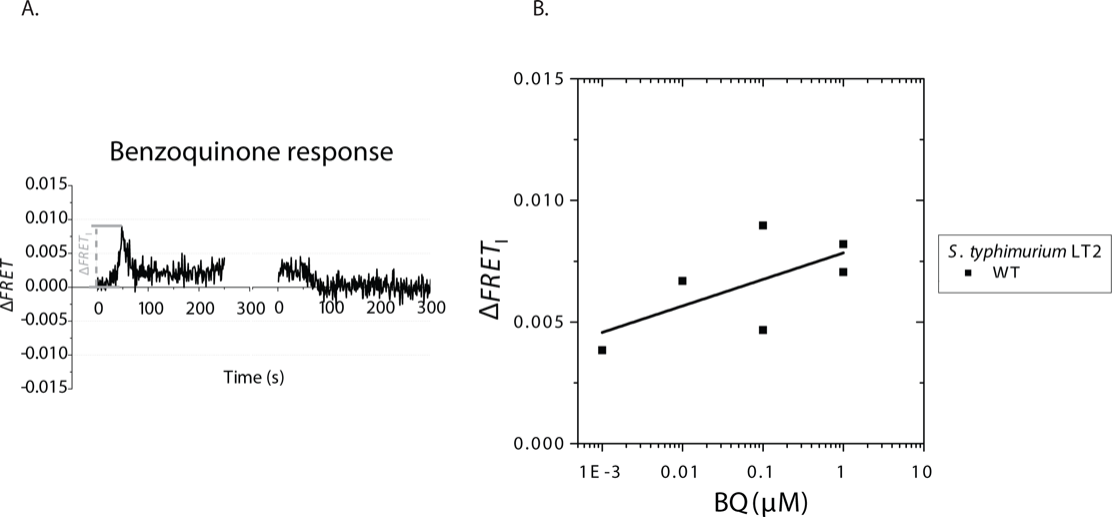
Repellent response to BQ of *S*. *typhimurium* LT2. **(A)** Typical time series of addition (left) and removal (right) of BQ (0.1 μM) in WT *S*. *typhimurium* LT2. BQ is added and removed at times 0. **(B)** Initial amplitudes of the FRET response (Δ*FRET*_I_) to BQ of WT *S*. *typhimurium* are plotted as a function of the BQ concentration.

Interestingly, benzoquinone (BQ)–the oxidized form of hydroquinone (HQ)–triggered similar responses as CySS: both components elicited a repellent response upon a step addition (the FRET level increases), followed by an imperfect adaptation (Fig 3). Moreover, responses to both compounds were detected even at 1 nM, and the dose-response dependences for both CySS and BQ appear to scale proportionately with the logarithm of the concentration over the tested range (Fig 3). We were not able to test BQ concentrations larger than 1 μM because responses started looking atypical for chemotactic kinase responses (*i*.*e*., an increase / decrease in both YFP and CFP channels; see Material and Methods), which could be due to the high toxicity of BQ to bacteria [39].

The reduced form, HQ, also elicited repellent responses, but only for concentrations ≥100 μM (S2 Fig), which had the same atypical character as the responses observed for high BQ concentrations. Due to the toxicity of HQ at high concentrations [39], we have not explored the HQ responses above 100 μM further. The observation that HQ does not trigger an attractant response suggests that the opposite responses to the Cys / CySS redox pair are not generalized redox responses.

### The sign of the response to mixed Cys / CySS solutions depends on their redox potential

The dose-response measurements in *S*. *typhimurium* LT2 *mcpB* / *C* knockout strains (Fig 2) suggested the existence of McpB / C-independent CySS-sensing mechanism in *S*. *typhimurium* LT2, which magnitude scales linearly with the logarithm of CySS concentration over the whole tested range of concentrations 20 nm–500 μM.

Due to the similar responses at nanomolar concentrations to CySS and BQ (Figs 2B and 3)–two oxidized compounds under the same aerobic conditions–we further explored the potential of Cys / CySS detection being relevant in redox taxis. CySS is a dimeric amino acid, formed by oxidation of the sulfhydryl (-SH) groups of two Cys monomers, leading to the formation of a disulfide bridge (-S-S-) by a redox reaction:

$$cysteine+cysteine+\frac{1}{2}O_{2}⇄cystine+H_{2}O$$

In order to evaluate the redox-dependence of the chemotactic response to Cys / CySS redox pair, we probed the response of wild type *S*. *typhimurium* LT2 to defined mixed solutions of Cys and CySS at different ratios (we accounted for 1% interconversion of Cys to CySS in all Cys solutions; see Fig 1, *Inset*).

**Fig 4:**
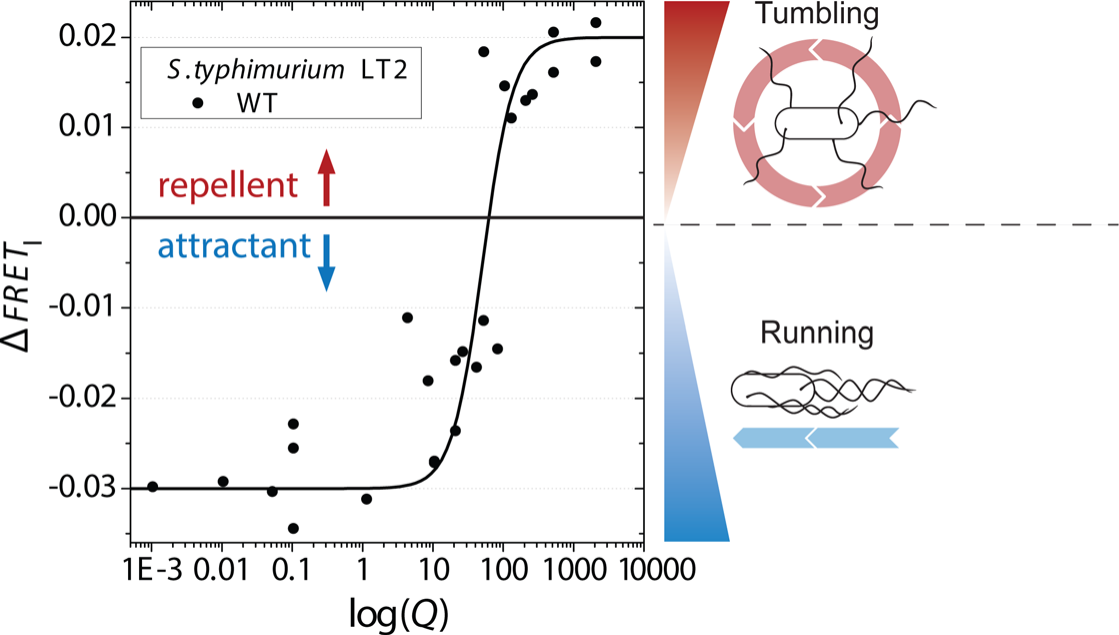
Response of *S*. *typhimurium* to mixed Cys / CySS solutions. Dependence of the initial amplitude of the FRET response, Δ*FRET*_I_, of WT *S*. *typhimurium* LT2 to the redox potential expressed in units of log Q, where Q is the reaction quotient of Cys / CySS interconversion. A Hill equation fit (with Hill coefficient of 2 and half-maximum of 51) is shown for guidance of the eye. The grey line at 0 divides attractant (negative) and repellent (positive) responses. Artist representations of a bacterium exhibiting an attractant response (“run”) and repellent response (“tumble”) are shown on the right.

We plotted the initial amplitude of the FRET response, Δ*FRET*_I_, for each Cys / CySS mixed solution against *log Q*, where *Q* is the reaction quotient of Cys / CySS interconversion reaction, *i*.*e*. $Q=\frac{\left[CySS\right]}{{\left[Cys\right]}^{2}}$ (Fig 4; Cys concentration is squared because two molecules participate in each oxidation reaction). The values of *log Q* are proportional to the changes in the redox potentials of the Cys / CySS solutions, which can be determined by the Nernst equation:

$$E_{h}=E_{0}+2.3\frac{RT}{nF}log\;Q,$$

where *E*_h_ is the redox potential of the solution, *E*_0_ is the standard redox potential of the Cys / CySS redox reaction, *R* is the gas constant, *T* is the absolute temperature, *n* is the number of moles of electrons transferred in the half redox reaction, *F* is the Faraday constant and *Q* is the reaction quotient of the redox reaction. For the Cys / CySS redox pair under the conditions of our experiments (room temperature, neutral pH), the redox potential (V) is:

$$E_{h}=-250+30log\;Q$$

For values of *log Q* < 100 the FRET efficiency was found to decrease, indicating an attractant response. For values of *log Q* > 100 the FRET efficiency was found to increase, indicating a repellent response. Thus, Cys / CySS mixed solutions appeared to elicit either an attractant or a repellent response depending on the value of *log Q*, *i*.*e*. the estimated redox potential of the solutions. Attractant response was observed for more reducing conditions, and repellent response was observed for more oxidizing conditions.

## Discussion

Motile bacteria often implement more than one pathway of sensing in order to orient in a gradient and find an optimal environmental niche. For example, thermotaxis and pH taxis responses both implement different receptors for sensing positive or negative changes from the optimal temperature and pH respectively [6, 22]. In this study, we found that the response of *S*. *typhimurium* LT2 to the Cys / CySS redox pair is mediated by different pathways. We confirmed that Tsr / Tar sense the reduced form (Cys) as an attractant and presented dose-response curves based on the changes in chemotactic signaling (Fig 1). In the case of CySS, we found an unexpected McpB / C- independent response starting at concentrations as low as 20 nM (Fig 2B). This is in contrast to previous measurements of the FRET response of *S*. *typhimurium* 14028, where the response to CySS was abolished in Δ*mcpB* Δ*mcpC* cells suggesting McpB / C-dependence [17].

LT2 and 14028 strains shared a common ancestor about 3000 to 9000 years ago, and the 14028 strain is more virulent [40, 41]. Their genomes are very similar (>98%) and the alignment of chemotaxis receptor nucleotide sequences shows no difference between the two strains. However, since the divergence with the virulence-attenuated LT2, the highly virulent 14028 strain accumulated 10% more base substitutions (spread genome-wide, primarily in non-synonymous sites) [41]. In light of this, it should be noted that different strains of one species can have up to a 100-fold difference in the activity of certain enzyme [42]. Therefore, differences among the 14028 and LT2 strains could explain the difference in the dependence on McpB / McpC in CySS detection.

The shape of the dose-response dependence of *S*. *typhimurium*’s response to CySS is atypical for receptor-ligand binding: the magnitude of the response scaled linearly with the logarithm of the CySS concentration over a very broad concentration range (20 nM–500 μM). We propose that these responses could be part of a redox-sensing pathway, based on our observations of similar responses to another oxidized compound, BQ (Fig 3). Redox-dependent inversion of the sign of the response to Cys / CySS mixed solutions (Fig 4) further suggests that a redox-dependent CySS-sensing pathway might exist. The redox responses in *S*. *typhimurium* could be mediated by Aer as shown for *E*. *coli* [43]. However, knocking-out *aer* did not show to have a major influence on the response to CySS in *S*. *typhimurium* (S1 Fig). Other redox-sensing receptors might exist: there are three *S*. *typhimurium* chemoreceptors that do not exist in *E*. *coli* (Tcp, Tip and the cytosolic receptor McpA, where the function of McpA and Tip is unknown).

It has been shown previously that redox molecules, such as substituted quinones, elicit redox taxis responses: in a spatial redox gradient, bacteria migrate towards a preferred redox potential, and these responses depend mainly on the Aer receptor in *E*. *coli* [7, 43]. In a study, the oxidized forms of quinone redox pairs were sensed as repellents under aerobic conditions [7]. Our FRET experiments, which were also performed under aerobic conditions, confirmed the latter observation: the oxidized compound BQ elicited a repellent response (Fig 3). The reduced form, HQ also elicited repellent responses but only for concentrations ≥100 μM (S2 Fig). Because of the atypical responses to BQ and HQ at high concentrations, which may result from their high toxicity to some bacteria [39], we have not explored these quinone responses further. However, our results with the HQ / BQ redox pair suggest that the opposite responses to the Cys / CySS redox pair are not generalized redox responses, and may be specific to Cys and CySS.

For all the tested strains and conditions, the chemotaxis adaptation to CySS and BQ in *S*. *typhimurium* LT2 and 14028 is imperfect. Neumann et al [44] showed that the imperfect adaptation to chemoeffectors has limited effect on cell population movement in a gradient but reduces the accumulation at high concentrations of the chemoeffectors. The elevated tumbling rate after incomplete adaptation helps the cells to keep exploring their surroundings and increase their chance to find more beneficial environment.

### What could be the physiological relevance of the multiple pathways of response to the Cys / CySS redox pair in *S*. *typhimurium*?

*Salmonella* actively cycles through host (e.g. human, animals) and non-host (e.g. soil, water) environments [45]. The physicochemical properties (e.g. pH, temperature, redox potential), as well as amino acid concentrations could be very different in these habitats. Therefore the ability to sense a wide range of conditions may be beneficial for *Salmonella*’s cyclic lifestyle (host–non-host environment–new host etc).

The plasma of a human in fasting state contains more CySS than Cys (concentrations ~42 μM and ~33 μM respectively) [46]. However, after CySS has been transported into various tissues it becomes reduced to Cys, and endogenously synthesized Cys would probably also remain in the reduced state [47]. Therefore it is likely that concentrations of Cys are higher within tissues.

*S*. *typhimurium* uses the amino acid Cys, a sulphur containing nutrient, in various metabolic pathways. Yang et al., [48] found that the utilization of an amino acid correlates with the attractant response in *E*. *coli*, where the response to Cys was among the strongest.

Apart from the metabolic importance of Cys, redox potential is an environmental factor that strongly affects cellular metabolism.I It has been hypothesized that redox taxis could guide bacterial species to niches with optimal redox conditions for different metabolic processes, such as hydrogen utilization and nitrogen fixation [7]. In this study we showed that the opposite responses to Cys and CySS might provide a mechanism for *S*. *typhimurium* to find optimal redox conditions.

Moreover, highly oxidizing conditions lead to the production of free oxygen radicals and oxidative stress, *i*.*e*. damage to DNA, lipids and proteins [49], and therefore can be detrimental for cells. Creating an oxidative microniche surrounding the macrophage cells is a strategy commonly used by the immune system of the host [50, 51], and an active mechanism of avoiding such microenvironments could promote the survival of *S*. *typhimurium* in the host. Previously it was proposed that such mechanism could allow *S*. *typhimurium* 14028 to escape from damage-inducing oxidative environments [17]. In this study we support this hypothesis by showing opposite responses to Cys and CySS of *S*. *typhimurium* LT2 and a transition point from attractant to repellent response based on the estimated redox potential of Cys / CySS mixed solutions.

Another advantage of having several pathways for Cys / CySS sensing is that it could allow bacteria to adjust their “preference” to match the requirements of the cells, by changing the expression levels of some of the chemoreceptors, as observed for pH taxis and thermotaxis [6, 22].

Although little is known about the habitat of the enteric bacteria in the gut, it is unlikely that steady amino acid / redox gradients are formed in the lumen. However, close to the wall of the intestine, there is a mucus layer consisting of glycoproteins (mucins), proteins, carbohydrates and lipids [52, 53]. The composition and rapid turnover of the mucus suggests the formation of amino acid / peptide gradients. The role of the mucus layer in protecting the underlying epithelial cells from contact with the luminal gut bacteria remains largely unknown [54].

We hypothesize that chemotaxis response to Cys / CySS might guide bacteria through the mucus layer towards the epithelial layer, facilitating systemic infections. Some of the mucins are Cys rich, and Cys gets crosslinked by disulfide bridges, giving the gel structure of the mucus layer, which prevents bacteria from penetrating the layer [55]. Defects in the crosslinking of the mucus, e.g. created by locally reducing conditions, might provide a path for systemic invasion of the bacteria. The opposite responses to the Cys / CySS redox pair might therefore improve the ability of pathogenic *S*. *typhimurium* strains to find such crosslinking defects in the structure of the mucus layer, and facilitate systemic infections [56].

Future studies of the behavior of *S*. *typhimurium* in defined Cys / CySS microenvironments and animal models could shed light on the relevance of Cys / CySS sensing for *S*. *typhimurium* survival and pathogenesis.

## Supporting Information

S1 Data Manuscript data. (XLSX)

S1 Fig Repellent response to CySS of Δ*aer* Δ*mcpC S*. *typhimurium* LT2. Typical time series of addition and removal of CySS (100 μM) in Δ*aer* Δ*mcpC S*. *typhimurium* LT2. Arrows indicate the times of addition and removal of CySS.
(TIF)

S2 Fig Repellent response to BQ and HQ of *S*. *typhimurium* LT2. Initial amplitudes of the FRET response (Δ*FRET*_I_) to BQ (black squares) and HQ (green circles) of WT *S*. *typhimurium* are plotted as a function of the BQ and HQ concentration.
(TIF)

## References

[1] Adler J. Chemoreceptors in bacteria. Science 166 1969;166(3913):1588 4902679 10.1126/science.166.3913.1588

[2] Adler J, Hazelbauer GL, Dahl MM. Chemotaxis toward sugars in Escherichia coli. J Bacteriol. 1973;115(3):824 4580570 10.1128/jb.115.3.824-847.1973 PMC246327

[3] Englert DL, Adase CA, Jayaraman A, Manson MD. Repellent taxis in response to nickel ion requires neither Ni2+ transport nor the periplasmic NikA binding protein. J Bacteriol. 2010;192(10):2633 10.1128/JB.00854-09 20233931 PMC2863559

[4] Kihara M, Macnab RM. Cytoplasmic pH mediates pH taxis and weak-acid repellent taxis of bacteria. J Bacteriol. 1981;145(3):1209–21. 7009572 10.1128/jb.145.3.1209-1221.1981 PMC217121

[5] Bibikov SI, Biran R, Rudd KE, Parkinson JS. A signal transducer for aerotaxis in Escherichia coli. J Bacteriol 179. 1997;179(12):4075 9190831 10.1128/jb.179.12.4075-4079.1997 PMC179224

[6] Salman H, Libchaber A. A concentration-dependent switch in the bacterial response to temperature. Nat Cell Biol 9. 2007;9(9):1098 17694049 10.1038/ncb1632

[7] Bespalov VA, Zhulin IB, Taylor BL. Behavioral responses of Escherichia coli to changes in redox potential. Proc Natl Acad Sci U S A. 1996;93(19):10084 8816755 10.1073/pnas.93.19.10084 PMC38340

[8] Sourjik V, Wingreen NS. Responding to chemical gradients: bacterial chemotaxis. Curr Opin Cell Biol. 2012;24(2):262 10.1016/j.ceb.2011.11.008 22169400 PMC3320702

[9] Wadhams GH, Armitage JP. Making sense of it all: bacterial chemotaxis. Nat Rev Mol Cell Biol. 2004;5(12):1024 15573139 10.1038/nrm1524

[10] Lux R, Munasinghe VR, Castellano F, Lengeler JW, Corrie JE, Khan S. Elucidation of a PTS-carbohydrate chemotactic signal pathway in Escherichia coli using a time-resolved behavioral assay. Mol Biol Cell. 1999;10(4):1133–46. 10198062 10.1091/mbc.10.4.1133 PMC25240

[11] Hazelbauer GL, Adler J. Role of the Galactose Binding Protein in Chemotaxis of Escherichia coli toward Galactose. Nat New Bio. 1971;230(101–104). 10.1038/newbio230101a0 4927373

[12] Lengeler JW, Jahreis K. Phosphotransferase systems or PTS as carbohydrate transport and as signal transduction systems In: Konings WN, Kaback HR, Lolkema JS, editors. Handbook of Biological Physics. Vol. 2 Amsterdam: Elsevier Science; 1996.

[13] Adler J. Chemotaxis in bacteria. Science. 1966;153(3737):708–16. 4957395 10.1126/science.153.3737.708

[14] Hazelbauer GL, Falke JJ, Parkinson JS. Bacterial chemoreceptors: high-performance signaling in networked arrays. Trends Biochem Sci. 2008;33(1):9 10.1016/j.tibs.2007.09.014 18165013 PMC2890293

[15] Briegel AL, X., Bilwes AM, Hughes KT, Jensen GJ, Crane BR. Bacterial chemoreceptor arrays are hexagonally packed trimers of receptor dimers networked by rings of kinase and coupling proteins. Proc Natl Acad Sci U S A. 2012;109(10):3766–71. 10.1073/pnas.1115719109 22355139 PMC3309718

[16] Berg HC. Motile Behavior of Bacteria. Physics Today. 2000;January 2000:24–9.

[17] Lazova MD, Butler MT, Shimizu TS, Harshey RM. Salmonella chemoreceptors McpB and McpC mediate a repellent response to L-cystine: a potential mechanism to avoid oxidative conditions. Mol Microbiol. 2012;84(4):697–711. 10.1111/j.1365-2958.2012.08051.x 22486902 PMC4285363

[18] Kondoh H, Ball CB, Adler J. Identification of a methyl-accepting chemotaxis protein for the ribose and galactose chemoreceptors of Escherichia coli. Proc Natl Acad Sci U S A. 1979;76(1):260–4. 370826 10.1073/pnas.76.1.260 PMC382918

[19] Mowbray SL, Koshland DEJ. Additive and independent responses in a single receptor: aspartate and maltose stimuli on the tar protein. Cell. 1987;50(2):171–80. 3297352 10.1016/0092-8674(87)90213-3

[20] Hedblom ML, Adler J. Chemotactic response of Escherichia coli to chemically synthesized amino acids. J Bacteriol. 1983;155(3):1463 6350273 10.1128/jb.155.3.1463-1466.1983 PMC217852

[21] Edwards JC, Johnson MS, Taylor BL. Differentiation between electron transport sensing and proton motive force sensing by the Aer and Tsr receptors for aerotaxis. Mol Microbiol. 2006;62(3):823–37. 16995896 10.1111/j.1365-2958.2006.05411.x PMC1858650

[22] Yang Y, Sourjik V. Opposite responses by different chemoreceptors set a tunable preference point in Escherichia coli pH taxis. Mol Microbiol. 2012;62(3):823–37. 10.1111/mmi.12070 23078189

[23] Lane MC, Lloyd AL, Markyvech TA, Hagan EC, Mobley HL. Uropathogenic Escherichia coli strains generally lack functional Trg and Tap chemoreceptors found in the majority of E. coli strains strictly residing in the gut. J Bacteriol. 2006;188(15):5618–25. 16855252 10.1128/JB.00449-06 PMC1540019

[24] Sourjik V, Berg HC. Functional interactions between receptors in bacterial chemotaxis. Nature 428. 2004;428(6981). 10.1038/nature02406 15042093

[25] Datsenko KA, Wanner BL. One-step inactivation of chromosomal genes in Escherichia coli K-12 using PCR products. Proc Natl Acad Sci U S A. 2000;97(12):6640 10829079 10.1073/pnas.120163297 PMC18686

[26] Yuan J, Berg HC. Resurrection of the flagellar rotary motor near zero load. Proc Natl Acad Sci U S A. 2008;105(4):1182–5. 10.1073/pnas.0711539105 18202173 PMC2234112

[27] Kelln RA, Lintott LG. Construction of plasmid-free derivatives of Salmonella typhimurium LT2 using temperature-sensitive mutants of pKZ1 for displacement of the resident plasmid, pSLT. Mol Gen Genet. 1990;222:438–40. 2274042 10.1007/BF00633852

[28] Sourjik V, Vaknin A, Shimizu TS, Berg HC. In vivo measurement by FRET of pathway activity in bacterial chemotaxis. Methods Enzymol. 2007;423:365–91. 17609141 10.1016/S0076-6879(07)23017-4

[29] Berg HC, Block SM. A miniature flow cell designed for rapid exchange of media under high-power microscope objectives. J Gen Microbiol. 1984;130(11):2915 6396378 10.1099/00221287-130-11-2915

[30] Shimizu TS, Tu Y, Berg HC. A modular gradient-sensing network for chemotaxis in Escherichia coli revealed by responses to time-varying stimuli. Mol Syst Biol. 2010;382 10.1038/msb.2010.37 20571531 PMC2913400

[31] Sourjik V, Berg HC. Receptor sensitivity in bacterial chemotaxis. Proc Natl Acad Sci U S A. 2002;99(1):123 11742065 10.1073/pnas.011589998 PMC117525

[32] Lazova MD, Ahmed T, Bellomo D, Stocker R, Shimizu TS. Response rescaling in bacterial chemotaxis. Proc Natl Acad Sci U S A. 2011;108(33):13870 10.1073/pnas.1108608108 21808031 PMC3158140

[33] Neidhardt FCeic. Escherichia coli and Salmonella: Cellular and Molecular Biology. Washington DC: ASM; 1996.

[34] McClelland M, Sanderson KE, Spieth J, Clifton SW, Latreille P, Courtney L, et al Complete genome sequence of Salmonella enterica serovar Typhimurium LT2. Nature. 2001;413(6858):852–6. 11677609 10.1038/35101614

[35] Dewey DL, Beecher J. Interconversion of Cystine and Cysteine induced by X-rays. Nature. 1965;206:1369–70. 5838255 10.1038/2061369a0

[36] Falke JJ. Cooperativity between bacterial chemotaxis receptors. Proc Natl Acad Sci U S A. 2002;99(10):6530 12011417 10.1073/pnas.112214199 PMC124436

[37] Stock J. Sensitivity, cooperativity and gain in chemotaxis signal transduction. Trends Microbiol. 1999;7(1):1 10068988 10.1016/s0966-842x(98)01429-2

[38] Prinz H. Hill coefficients, dose-response curves and allosteric mechanisms. J Chem Biol. 2010;3(1):37–44. 10.1007/s12154-009-0029-3 19779939 PMC2816740

[39] Trevors JT, J. B. Toxicity of Benzoquinone and Hydroquinone in Short-Term Bacterial Bioassays Bull Environm Contam Toxicol. 1980;25(672–675). 10.1007/BF01985590 6777005

[40] Garcia-Quintanilla M, Casadesus J. Virulence plasmid interchange between strains ATCC 14028, LT2, and SL1344 of Salmonella enterica serovar Typhimurium. Plasmid. 2011;65(2):169 10.1016/j.plasmid.2010.12.001 21145349

[41] Jarvik T, Smillie C, Groisman EA, Ochman H. Short-term signatures of evolutionary change in the Salmonella enterica serovar typhimurium 14028 genome. J Bacteriol. 2010;192(2):560 10.1128/JB.01233-09 19897643 PMC2805332

[42] Burne RA, Zeng L, Ahn SJ, Palmer SR, Liu Y, Lefebure T, et al Progress dissecting the oral microbiome in caries and health. Adv Dent Res. 2012;24:77–80. 10.1177/0022034512449462 22899685 PMC3420362

[43] Rebbapragada A, Johnson MS, Harding GP, Zuccarelli AJ, Fletcher HM, Zhulin IB, et al The Aer protein and the serine chemoreceptor Tsr independently sense intracellular energy levels and transduce oxygen, redox, and energy signals for Escherichia coli behavior. Proc Natl Acad Sci U S A. 1997;94(20):10541–6. 9380671 10.1073/pnas.94.20.10541 PMC23396

[44] Neumann S, Vladimirov N, Krembel AK, Wingreen NS, Sourjik V. Imprecision of adaptation in Escherichia coli chemotaxis. PLoS One. 2014;9(1). 10.1371/journal.pone.0084904 PMC3885661 24416308

[45] Winfield MD, Groisman EA. Role of nonhost environments in the lifestyles of Salmonella and Escherichia coli. Appl Environ Microbiol. 2003;69(7):3687–94. 12839733 10.1128/AEM.69.7.3687-3694.2003 PMC165204

[46] Brigham MP, Stein WH, Moore S. The concentrations of cysteine and cystine in human blood plasma. J Clin Invest. 1960;39(11):1633–8. 16695834 10.1172/JCI104186 PMC293403

[47] Crawhall JC, Segal S. The intracellular ratio of cysteine and cystine in various tissues. Biochem J. 1967;105(2):891–6. 5584026 10.1042/bj1050891 PMC1198391

[48] Yang YM, Pollard A, Höfler C, Poschet G, Wirtz M, Hell R, et al Relation between chemotaxis and consumption of amino acids in bacteria. Mol Microbiol. 2015;96(6):1272–82. 10.1111/mmi.13006 25807888 PMC5008178

[49] Rosner JL, Storz G. Regulation of bacterial responses to oxidative stress. Curr Top Cell Regul. 1997;35:163 9192180 10.1016/s0070-2137(97)80007-6

[50] Ishii T, Itoh K, Sato H, Bannai S. Oxidative stress-inducible proteins in macrophages. Free Radic Res. 1999;31(4):351 10517540 10.1080/10715769900300921

[51] McGhie EJ, Brawn LC, Hume PJ, Humphreys D, Koronakis V. Salmonella takes control: effector-driven manipulation of the host. Curr Opin Microbiol. 2009;12(1):117–24. 10.1016/j.mib.2008.12.001 19157959 PMC2647982

[52] Corazziari ES. Intestinal mucus barrier in normal and inflamed colon. J Pediatr Gastroenterol Nutr. 2009;48(Suppl 2):S54–5. 10.1097/MPG.0b013e3181a117ea 19300126

[53] Swidsinski A, Loening-Baucke V, Theissig F, Engelhardt H, Bengmark S, Koch S, et al Comparative study of the intestinal mucus barrier in normal and inflamed colon. Gut. 2007;56(3):343–50. 16908512 10.1136/gut.2006.098160 PMC1856798

[54] Corfield AP, Myerscough N, Longman R, Sylvester P, Arul S, Pignatelli M. Mucins and mucosal protection in the gastrointestinal tract: new prospects for mucins in the pathology of gastrointestinal disease. Gut. 2000;47(4):589–94. 10986224 10.1136/gut.47.4.589 PMC1728059

[55] Kim YS, Ho SB. Intestinal goblet cells and mucins in health and disease: recent insights and progress. Curr Gastroenterol Rep. 2010;12(5):319–30. 10.1007/s11894-010-0131-2 20703838 PMC2933006

[56] Lindén SK, Florin TH, McGuckin MA. Mucin dynamics in intestinal bacterial infection. PLoS One. 2008;3(12):e3952 10.1371/journal.pone.0003952 19088856 PMC2601037

