# Supplementary figures and images for: Dose-Response Analysis of Chemotactic Signaling Response in Salmonella typhimurium LT2 upon Exposure to Cysteine / Cystine Redox Pair
Source: PLoS One. 2016 Apr 7;11(4):e0152815. doi: 10.1371/journal.pone.0152815 (PMC4824473; doi:10.1371/journal.pone.0152815)

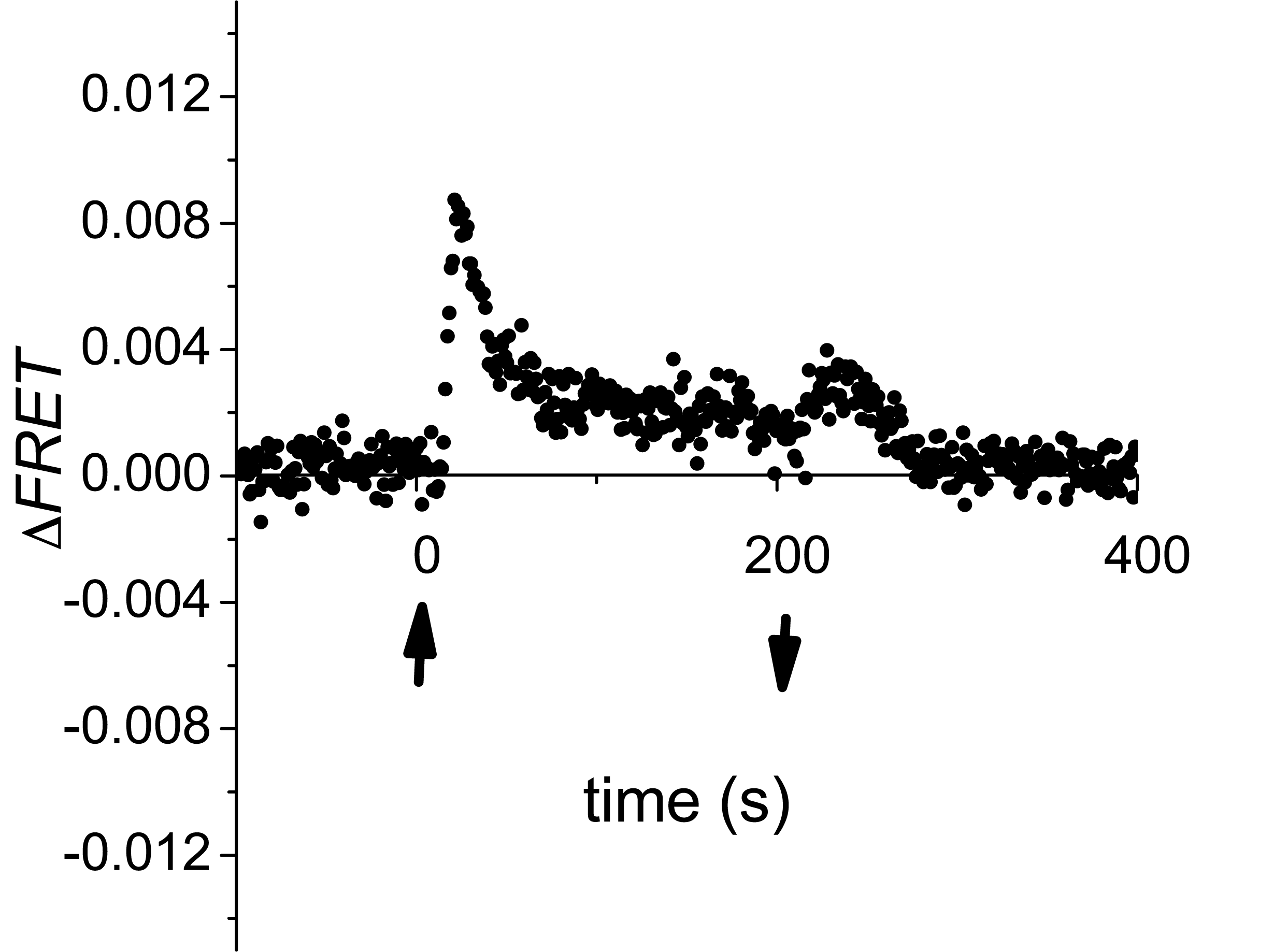

Supplement: S1 Fig — Typical time series of addition and removal of CySS (100 μM) in Δaer ΔmcpC S. typhimurium LT2. Arrows indicate the times of addition and removal of CySS. (TIF) [file pone.0152815.s002.tif]

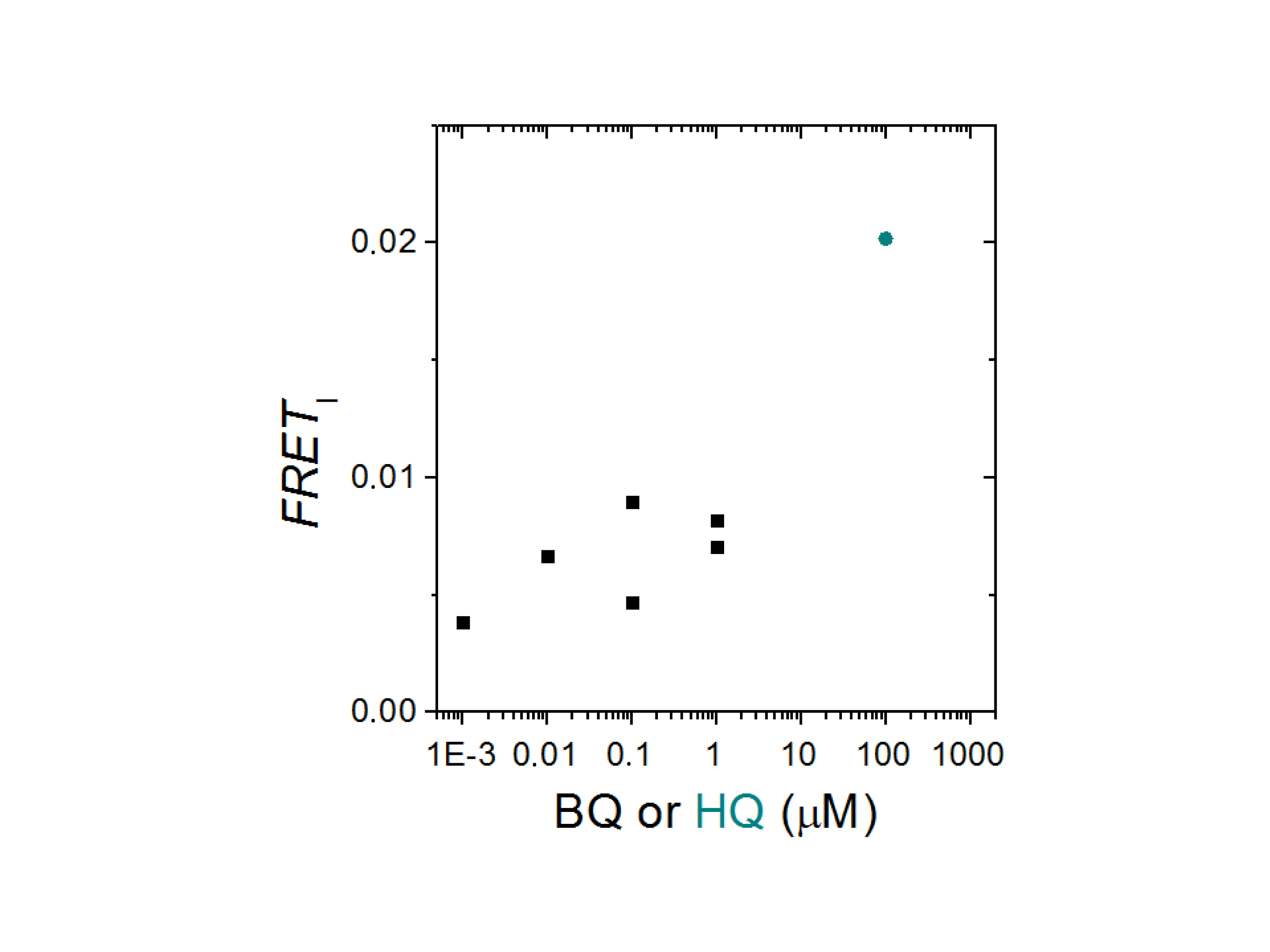

Supplement: S2 Fig — Initial amplitudes of the FRET response (ΔFRETI) to BQ (black squares) and HQ (green circles) of WT S. typhimurium are plotted as a function of the BQ and HQ concentration. (TIF) [file pone.0152815.s003.tif]
